# Disparities in outcomes among patients on lung transplantation waitlist in Saudi Arabia

**DOI:** 10.3389/ti.2026.17165

**Published:** 2026-10-01

**Authors:** Amjad Albatli, Ruqayyah Fadhel, Yehia Rabea Yousef, Rana Ahmed Saleh, Haifa Aldakhil, Yasmin Altwaijri, Faisal Albaiz, Khaled Alkattan, Waleed Saleh, Moheeb Alawwami, Amer Alzahrani, Husna Thalib, Bedour Almudaiheem, Kris Marquez, Rayid Abdulqawi

**Affiliations:** 1 Lung Health Centre Department, Organ Transplant Centre of Excellence, King Faisal Specialist Hospital and Research Centre, Riyadh, Saudi Arabia; 2 Department of Chest Diseases, Faculty of Medicine, Ain Shams University, Egypt; 3 Research Laboratories Department, Research and Innovation, King Faisal Specialist Hospital and Research Centre, Riyadh, Saudi Arabia; 4 College of Medicine, Alfaisal University, Riyadh, Saudi Arabia; 5 Histocompatibility Lab, Kidney and Pancreas Health Centre Department, Organ Transplant Centre of Excellence, King Faisal Specialist Hospital and Research Centre, Riyadh, Saudi Arabia; 6 General Medicine Practice Program, Batterjee Medical College, Jeddah, Saudi Arabia; 7 Data Analytics Centre, Organ Transplant Centre of Excellence, King Faisal Specialist Hospital and Research Centre, Riyadh, Saudi Arabia

**Keywords:** access, disparity, lung transplant, lung transplant-allocation, Saudi Arabia, waitlist outcomes

Dear Editors,

The impact of lung transplantation is limited by scarcity of suitable donor lungs. Therefore, optimizing donor lung allocation is essential to ensure that patients with the greatest need and highest likelihood of benefit receive these organs. Studies reporting disparities in access to lung transplantation from regions outside North America and Europe are limited. In Saudi Arabia, until early 2024, King Faisal Specialist Hospital and Research Centre (KFSHRC) served as the only center that performed lung transplants; during this period, donor lung allocation was determined by the transplant team based on clinical judgment. In 2024, a second lung transplant center was established, and the Composite Allocation Score (CAS) was nationally adopted to determine priority for receiving a lung transplant.

This unique pre-CAS period provided an opportunity to report a national baseline of waitlist outcomes and disparities in access to transplantation in Saudi Arabia. Therefore, we conducted a retrospective cohort study that included all consecutive adult patients (aged ≥18 years) listed for a first-time lung-only transplant at KFSHRC between January 1, 2014, and December 31, 2023, with follow-up data until June 30, 2025. Competing risk regression was used to identify predictors of transplantation versus death or clinical deterioration.

In total, 368 patients were included (median age: 45 years; 52% male). Compared with male patients, females were significantly younger (43.0 vs. 46.0 years; *P* = 0.009) and had significantly higher rates of sensitization (calculated panel reactive antibody [cPRA] ≥50%: 28.1% vs. 9.5%, *P* < 0.001; cPRA ≥80%: 19.1% vs. 2.6%; *P* < 0.001). The most common blood groups were type O (48.1%), and the most common indication was restrictive lung disease (60.9%) (Supplementary Table S1).

A total of 257 patients (70%) underwent transplantation, of which 159 were male (62%) and 98 were female (38%). Further, 13.6% died while on the waitlist, 7.9% were removed from the waitlist for other reasons, and 8.7% remained on the list. The unadjusted 1-year cumulative incidence rates of transplantation, mortality, and waitlist removal were 63.5%, 9.2%, and 5.0%, respectively (Figure 1). The median time to transplant was 118 days (IQR: 50–194 days), and was similar for females (121 days; IQR: 42–246) and males (116 days; IQR: 51–181) (*P* = 0.705) (Supplementary Table S2). In the multivariable Fine‒Gray competing risk analysis, compared with females, male candidates had a 70% greater likelihood of undergoing transplantation (subdistribution hazard ratio [SHR]: 1.7; 95% CI: 1.23–2.36; *P* = 0.001). In addition, blood group O was associated with a 43% lower likelihood of transplantation (SHR: 0.43; 95% CI: 0.33–0.57; *P* < 0.001) and a 2.2-fold greater risk of waitlist mortality (SHR: 2.2; 95% CI: 1.05–4.64; *P* = 0.038) (Supplementary Table S3).

**FIGURE 1:**
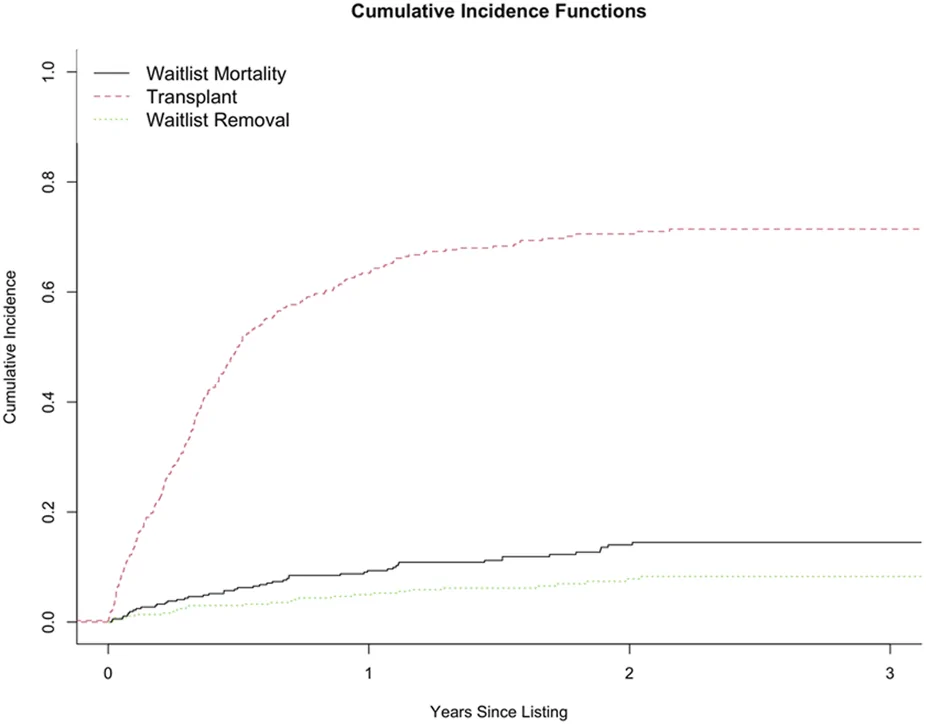
Cumulative incidence of competing outcomes among patients listed for lung transplantation.

Following transplantation, female recipients had significantly longer durations of mechanical ventilation and hospital and ICU stays as well as more frequent postoperative ECMO use compared with males; 90-day mortality was comparable between both sexes (Supplementary Table S4). The graft survival probabilities were 86% at 1 year, 72% at 3 years, and 55% at 5 years (Supplementary Figure S1). In terms of donors, more than three-fourths were male (78.6%) (median age: 41 years).

Our findings revealed a relatively young candidate population in Saudi Arabia, with a disease distribution distinct from that reported in Western registries [1]. In addition, we found that reduced transplant access among female candidates and those with blood group O. These results highlight the importance of understanding regional variation in transplant candidacy and suggest that allocation policies should consider the local demographic and biological characteristics.

Our finding that females are less likely to undergo lung transplantation than males is consistent with that in the literature, including the Organ Procurement and Transplantation Network-based analysis by Riley and Lascano [2] and findings from France [3]. This disparity was after adjusting for the significantly higher rates of sensitization and shorter stature among female candidates, suggesting systemic barriers beyond clinical or anthropometric differences may have contributed to reduced access; however, the influence of other unmeasured factors cannot be excluded. This persistent sex disparity underscores the need for targeted interventions to improve equity in transplant access. In unadjusted analyses, female recipients also experienced significantly worse early post-transplant outcomes despite comparable 90-day mortality, and this could possibly be due to donor–recipient size mismatch, which disproportionately affects females given their shorter stature relative to the predominantly male donor pool, and a higher HLA sensitization burden.

Saudi Arabia faces shortages for deceased donor organs, which is is substantially lower (at 4.0 million/population) than that of Spain (49.4 million/population) and the United States (48.0 million/population) [4]. Reported local barriers to deceased donation include confusion between brain death and coma, inconsistent ICU communication, and religious and cultural hesitancy, despite formal religious approval of organ donation [5]. These factors highlight the importance of ongoing national efforts such as the Saudi Center for Organ Transplantation Proactive Detection Program [6] and digital donor registration initiatives [7] to help expand the deceased donor pool.

In addition to these initiatives, future strategies that can be considered to address donor scarcity in Saudi Arabia include further broadening the use of extended-criteria donors [8] and donation after circulatory death [9]. A Gulf Cooperation Council countries-wide organ-sharing network (similar to Eurotransplant), *ex vivo* lung perfusion, and use of novel preservation technologies are other possible strategies that may reduce lung donor scarcity and preservation. In select patients, living-donor lobar lung transplantation [10] may represent another option, particularly given Saudi Arabia’s comparatively strong living-donation culture.

A limitation of this study was that due to the retrospective study design, it may be subject to unmeasured confounding factors. In addition, the generalizability of these findings to the current national context is limited by the recent establishment of a second lung transplant program and the adoption of the CAS system.

In conclusion, the current study found that there were disparities in access to lung transplantation by sex and blood group. The data from the current study provides a national pre-CAS benchmark for future studies to determine if implementation of a formal allocation system improves equity and timely access to and outcomes of transplantation in Saudi Arabia.

## Data Availability

The original contributions presented in the study are included in the article/Supplementary Material, further inquiries can be directed to the corresponding author.
