# Supplementary material for: Disparities in outcomes among patients on lung transplantation waitlist in Saudi Arabia
Source: Transpl Int. 2026 Oct 1;39:17165. doi: 10.3389/ti.2026.17165 (PMC13668760; doi:10.3389/ti.2026.17165)
Supplement: Supplementary file 1 [file Supplementaryfile1.docx]

**SUPPLEMENTARY TABLES**

**Supplementary Table 1.** Clinical characteristics of lung transplant candidates at the time of listing

|  | **Overall**, *N* = 368 | **Female**, *N* = 178 | **Male**, *N* = 190 | ***P*-value**^a^ |
| --- | --- | --- | --- | --- |
| **Age (years), median (IQR)** | 45.0 (33.0, 55.0) | 43.0 (31.0, 53.0) | 46.00 (34.0, 57.7) | 0.009 |
| **Height (cm), median (IQR)** | 160.2 (154.0, 168.0) | 154.0 (151.0, 159.0) | 167.0 (162.3, 172.0) | <0.001 |
| **Height <155 (cm), *n* (%)** | 100 (27.2) | 91 (51.1) | 9 (4.7) | <0.001 |
| **Weight (kg), median (IQR)** | 59.0 (45.4, 70.6) | 54.3 (41.8, 63.9) | 62.6 (49.6, 74.8) | <0.001 |
| **BMI (kg/m²), median (IQR)** | 21.9 (18.2, 26.7) | 22.1 (18.1, 27.1) | 21.8 (18.4, 26.2) | 0.686 |
| **BMI (kg/m²) ≤16, *n* (%)** | 44 (12.0) | 24 (13.5) | 20 (10.5) | 0.382 |
| **Blood Type, *n* (%)** |  |  |  | 0.619 |
| A | 105 (28.5) | 46 (25.8) | 59 (31.0) |  |
| AB | 13 (3.5) | 7 (3.9) | 6 (3.2) |  |
| B | 73 (19.9) | 39 (21.9) | 34 (17.9) |  |
| O | 177 (48.1) | 86 (48.3) | 91 (47.9) |  |
| **OPTN Diagnosis Group^b^, *n* (%)** |  |  |  | <0.001 |
| A | 82 (22.3) | 40 (22.5%) | 42 (22.1%) |  |
| B | 25 (6.8) | 22 (12.4%) | 3 (1.6%) |  |
| C | 37 (10.0) | 20 (11.2%) | 17 (8.9%) |  |
| D | 224 (60.9) | 96 (53.9%) | 128 (67.4%) |  |
| **cPRA ≥50%, *n* (%)** | 68 (18.5) | 50 (28.1%) | 18 (9.5%) | <0.001 |
| **cPRA ≥80%, *n* (%)** | 39 (10.6) | 34 (19.1%) | 5 (2.6%) | <0.001 |
| ^a^Wilcoxon rank sum test; Pearson's Chi-squared test; Fisher's exact test | | | | |
| ^b^Organ Procurement and Transplantation Network (OPTN) Diagnosis Group A: Noncystic fibrosis bronchiectasis (n=62), COPD (n=13), sarcoidosis (n=7). B: pulmonary arterial hypertension (n=25). C: cystic fibrosis (n=36), primary immunodeficiency (n=1). D: lung fibrosis (n=200), pulmonary chronic graft versus host disease (n=9), sarcoidosis with PA mean pressure >30 mmHg (n=6), pulmonary alveolar microlithiasis (n=5), Langerhans cell histiocytosis (n=2), idiopathic pulmonary hemosiderosis (n=1), obliterative bronchiolitis non-retransplant (n=1). | | | | |
| BMI, body mass index; cPRA, Calculated panel-reactive antibody | | | | |

**Supplementary Table 2**. Clinical and surgical characteristics of lung transplant recipients

|  | **Overall** (*N* = 257) | **Female** (*N* = 98) | **Male** (*N* = 159) | ***P*-value**^a^ |
| --- | --- | --- | --- | --- |
| **Age (years), median (IQR)** | 44.0 (33.0, 55.0) | 41.0 (29.2, 52.0) | 45.0 (34.0, 57.0) | 0.021 |
| **Height <155 (cm), *n* (%)** | 50 (19.5%) | 43 (43.9%) | 7 (4.4%) | <0.001 |
| **BMI (kg/m²), median (IQR)** | 21.6 (18.1, 26.3) | 21.2 (17.9, 26.6) | 21.70 (18.3, 26.1) | 0.793 |
| **BMI ≤16 (kg/m²), *n* (%)** | 32 (12.4) | 14 (14.3) | 18 (11.3) | 0.484 |
| **Blood Type, *n* (%)** |  |  |  | 0.101 |
| A | 87 (33.9) | 32 (32.7) | 55 (34.6) |  |
| AB | 11 (4.3) | 6 (6.1) | 5 (3.1) |  |
| B | 57 (22.2) | 28 (28.6) | 29 (18.2) |  |
| O | 102 (39.7) | 32 (32.7) | 70 (44.0) |  |
| **OPTN Diagnosis Group^b^, *n* (%)** |  |  |  | 0.136 |
| A | 56 (21.8) | 21 (21.4) | 35 (22.0) |  |
| B | 10 (3.9) | 7 (7.1) | 3 (1.9) |  |
| C | 31 (12.1) | 14 (14.3) | 17 (10.7) |  |
| D | 160 (62.3) | 56 (57.1) | 104 (65.4) |  |
| **Lung Transplant Surgery, *n* (%)** |  |  |  | 0.065 |
| Bilateral | 232 (90.6) | 93 (94.9) | 139 (88.0) |  |
| Single | 24 (9.4) | 5 (5.1) | 19 (12.0) |  |
| (missing) | 1 | 0 | 1 |  |
| **Waiting time to transplant (days), median (IQR)** | 118.0 (50.0, 194.0) | 121.0 (42.0, 246.0) | 116.0 (51.00, 181.5) | 0.705 |
| **Total ischemia time (minutes), median (IQR)** | 334.7 (270.0, 390.0) | 334.5 (282.5, 390.0) | 335.0 (269.0, 390.0) | 0.672 |
| (missing) | 13 | 5 | 8 |  |
| **Pre-transplant recipient location, *n* (%)** |  |  |  | 0.394 |
| Home | 209 (81.9) | 78 (80.4) | 131 (82.9) |  |
| Inpatient other than ICU | 22 (8.6) | 7 (7.2) | 15 (9.5) |  |
| ICU | 24 (9.4) | 12 (12.4) | 12 (7.6) |  |
| (missing) | 2 | 1 | 1 |  |
| **Bridge to transplant MV, *n* (%)** | 23 (9.0) | 10 (10.3) | 13 (8.2) | 0.573 |
| (missing) | 2 | 1 | 1 |  |
| **Bridge to transplant ECMO, *n* (%)** | 9 (3.5) | 6 (6.2) | 3 (1.9) | 0.088 |
| (missing) | 2 | 1 | 1 |  |
| **Intraoperative support, *n* (%)** |  |  |  | <0.001 |
| Cardiopulmonary bypass | 2 (0.8) | 0 (0.0) | 2 (1.3) |  |
| ECMO | 190 (76.3) | 88 (91.7) | 102 (66.7) |  |
| None | 57 (22.9) | 8 (8.3) | 49 (32.0) |  |
| (missing) | 8 | 2 | 6 |  |
| ^a^Wilcoxon rank sum test; Fisher's exact test; Pearson's Chi-squared test | | | | |
| ^b^Organ Procurement and Transplantation Network Diagnosis Group A: Noncystic fibrosis bronchiectasis (*n*=44), COPD (*n*=9), sarcoidosis (*n*=3). B: pulmonary arterial hypertension (*n*=10). C: cystic fibrosis (*n*=30), primary immunodeficiency (*n*=1). D: lung fibrosis (*n*=141), pulmonary chronic graft versus host disease (*n*=8), sarcoidosis with PA mean pressure >30 mmHg (*n*=5), pulmonary alveolar microlithiasis (*n*=4), Langerhans cell histiocytosis (*n*=1), obliterative bronchiolitis non-retransplant (*n*=1). | | | | |
| BMI, body mass index; MV, mechanical ventilation; ECMO, extracorporeal membrane oxygenation; ICU, intensive care unit | | | | |

**Supplementary Table 3.** Multivariable competing risks regression for waitlist mortality and transplantation

| **Variable** | **SHR (Death)** | **95% CI** | ***P*-value** | **SHR (Transplant)** | **95% CI** | ***P*-value** |
| --- | --- | --- | --- | --- | --- | --- |
| **Male (Ref: Female)** | 0.64 | 0.32,1.26 | 0.200 | 1.70 | 1.23,2.36 | 0.001 |
| **Age at Listing** | 1.00 | 0.98,1.03 | 0.870 | 0.99 | 0.98,1 | 0.110 |
| **Diagnosis Group B (Ref: A)** | 2.35 | 0.94,5.87 | 0.069 | 0.61 | 0.29,1.27 | 0.190 |
| **Diagnosis Group C (Ref: A)** | NE | - | - | 1.50 | 0.99,2.27 | 0.055 |
| **Diagnosis Group D (Ref: A)** | 1.05 | 0.51,2.14 | 0.900 | 0.89 | 0.64,1.26 | 0.520 |
| **cPRA ≥50%** | 1.37 | 0.67,2.78 | 0.390 | 0.69 | 0.46,1.01 | 0.057 |
| **Blood Group AB (Ref: A)** | 1.80 | 0.3,10.62 | 0.520 | 1.38 | 0.58,3.25 | 0.460 |
| **Blood Group B (Ref: A)** | 1.08 | 0.39,2.98 | 0.880 | 1.04 | 0.72,1.52 | 0.820 |
| **Blood Group O (Ref: A)** | 2.20 | 1.05,4.64 | 0.038 | 0.43 | 0.33,0.57 | <0.001 |
| **Height <155 cm** | 1.02 | 0.54,1.95 | 0.940 | 0.70 | 0.47,1.02 | 0.066 |

CI, Confidence interval; SHR, subdistribution hazard ratio; NE, not estimable due to zero events

**Supplementary Table 4**. Early post-transplant outcomes

|  | **Overall** (*N* = 257) | **Female** (*N* = 98) | **Male** (*N* = 159) | ***P*-value**^a^ |
| --- | --- | --- | --- | --- |
| **Ventilator days, median (IQR)** | 5.0 (2.0, 20.0) | 8.5 (2.0, 30.0) | 5.0 (2.0, 16.0) | 0.044 |
| **ICU days, median (IQR)** | 12.0 (6.0, 25.0) | 15.5 (8.7, 34.5) | 11.0 (5.0, 21.0) | 0.002 |
| (missing) | 3 | 2 | 1 |  |
| **Hospital stay (days), median (IQR)** | 32.0 (20.7, 53.0) | 37.0 (23.0, 64.0) | 29.0 (20.0, 44.0) | 0.012 |
| (missing) | 1 | 1 | 0 |  |
| **Need for ECMO, *n* (%)** | 54 (21.1%) | 30 (30.6%) | 24 (15.2%) | 0.003 |
| (missing) | 1 | 0 | 1 |  |
| **Need for tracheostomy, *n* (%)** | 86 (33.5%) | 38 (38.8%) | 48 (30.2%) | 0.156 |
| **90-day mortality, *n* (%)** | 17 (6.6%) | 7 (7.1%) | 10 (6.3%) | 0.789 |
| ^a^Pearson's Chi-squared test; Wilcoxon rank sum test | | | | |
| ICU, intensive care unit; ECMO, extracorporeal membrane oxygenation. | | | | |

**SUPPLEMENTARY FIGURE**

**Supplementary Figure 1:** Graft survival following lung transplantation
